# Ontogeny of Diet and Behavior of a Wild, Critically Endangered Lemur (*Indri indri*)

**DOI:** 10.1002/ajp.70107

**Published:** 2025-12-18

**Authors:** Giada Brunod, Federica Dellepiane, Daria Valente, Valeria Ferrario, Filippo Carugati, Valeria Torti, Jonah Ratsimbazafy, Cristina Giacoma, Marco Gamba, Chiara De Gregorio

**Affiliations:** ^1^ Department of Life Sciences and Systems Biology University of Torino Torino Italy; ^2^ Groupe d'Etude et de recherche sur les primates de Madagascar Antananarivo Madagascar; ^3^ Department of Psychology University of Warwick Coventry UK

**Keywords:** co‐feeding, coprophagy, development, feeding, infant, lemurs

## Abstract

The early developmental period plays a key role in primate behavioral outcomes. Understanding the behavioral ecology of infant indris (*Indri indri*) helps to identify resources needed during early developmental stages, the weaning process, and the role of mothers in developing infants' survival abilities. In this study, we investigated the behavioral and dietary development of wild *Indri indri* in Madagascar. We found that infants undergo significant behavioral transitions during their early growth, which reflect their maturation and adaptation to the environment. Play is critical in developing locomotion skills and acquiring knowledge about dietary preferences. Social interactions, initially centered on grooming with the mother, began to extend to other group members as early as 4 months of age, reflecting a shift from maternal care to broader social bonding. Additionally, territorial behaviors such as scent marking became more prominent in later month age classes when new behaviors, such as singing, emerged. We observed a co‐feeding relationship between young indris and their mothers that ceased around 1 year of age. Lauraceae was the most‐eaten plant family in the first 2 years of life, and we observed shifts in plant taxa and plant parts consumption with age. Milk consumption was not observed after 7 months of age. We also highlighted the presence of coprophagy and geophagy in indri infants, which were observed on several occasions consuming maternal feces and soil. These behaviors could play a role in maternal microbiota inoculation and toxin regulation, though further evidence is needed, even at a young age. Our work highlighted the dietary requirements and behavioral development of indri infants. These Critically Endangered lemurs have never been bred in captivity; our findings provide foundational data to inform future studies and potential conservation strategies.

## Summary

- Understanding the behavioral ecology of infant indris is critical to identify resources needed during early developmental stages, and for monitoring the health of individuals or populations of these critically endangered primates.
- We found that leaves are the most consumed food in the early stages, while fruit consumption rises noticeably after the first 3 months of life; milk consumption stopped after 7 months of age.
- We highlighted the presence of coprophagy and geophagy in infants, that were observed consuming maternal feces and soil: these behaviors might be critical to inoculate the mother's microbiota and regulating levels of food toxins, even at a young age.

## 1 Introduction

Primates are known for their prolonged dependence on their mothers due to the extended acquisition of diet preferences and social behavior. This dependent period is critical, during which young learn skills by exploring and interacting with others (Vochteloo et al. 1993). Their progression towards independence is marked by the ability to move, feed, and interact without being close to their mother (Southwick and Siddiqi 1974; Altmann 1980). Developing and changing behavioral patterns in animals can be classified into four stages: infancy, youth, adolescence, and maturity (Pereira and Altmann 1985). During infancy, the animal relies on its mother for food, protection, and safety (Baldwin and Baldwin 1977; Pereira and Altmann 1985). Weaning marks the transition to the youth stage, when the animal reduces contact with its mother and focuses on exploration, play, and social learning (Hinde and Spencer‐Booth 1967; Pereira and Altmann 1985). This phase is crucial for developing social relationships and acquiring behaviors (Pereira and Altmann 1985). Adolescence begins with puberty, marked by changes in aggressive and sexual behavior (especially in males), and often involves dispersal from the natal group, though this can also occur earlier in some species (Charles‐Dominique, 1977; Rijksen, 1978; Dawson, 1977). It ends with the attainment of reproductive capacity, marking the onset of maturity (Pereira and Altmann 1985).

In primates, social learning plays a central role in development, supported by their characteristically extended juvenile period (Whiten 2000; Joffe 1997). In orangutans (*Pongo* spp.), juveniles learn to process food and build nests by observing and imitating their mothers and sometimes their older siblings (Van Noordwijk et al. 2009; Jaeggi et al. 2010; Schuppli et al. 2016). For chimpanzees (*Pan troglodytes schweinfurthii*), young females spend much time observing their mothers' termite fishing, which helps them master the technique earlier than their brothers (Lonsdorf et al. 2004; Lonsdorf 2005, 2006). Similarly, female Japanese macaques (*Macaca fuscata*) learn grooming behavior by imitating the matriarch's movements. This behavior is also imitated by daughters and granddaughters, indicating that it has a learning purpose (Tanaka 1998). Furthermore, social learning can involve other group members as well. In the context of feeding, “co‐feeding” occurs when two or more individuals feed simultaneously and in close proximity (Rapaport and Brown 2008; Pimley et al. 2005; Nicolson 1987). This social behavior has been observed in various species. Young ring‐tailed lemurs tend to stay closer to the individuals near them when feeding on solid food than older group members (O'Mara and Hickey 2012; Ueno 2005). This behavior suggests that social learning plays a significant role in accelerating the learning process of primates.

*Indri indri* is a Critically Endangered lemur species according to the IUCN Red List of Threatened Species (King et al. 2020), endemic to the eastern rainforests of Madagascar. Indris are diurnal and arboreal primates, typically living in small monogamous and territorial family groups (Torti et al. 2017; Bonadonna et al. 2019, 2020). Females give birth to a single offspring every 2–3 years, and the species suffers from high infant mortality (Rolle et al. 2021), making each developmental stage potentially critical for survival. Their diet mainly consists of immature leaves, supplemented with fruit, flowers, seeds, and bark (Pollock 1975a; Britt et al. 2003; Powzyk and Mowry 2006), and includes about 139 plant species (Randrianarison et al. 2022). Behaviors such as geophagy have also been observed (Pollock 1975a; Powzyk 1997), potentially serving detoxifying or nutritional functions (Britt et al. 2003; Borruso et al. 2021). In captivity, breeding attempts have consistently failed, largely due to the challenges of replicating their natural diet (Petter et al. 1977; Britt et al. 2003). Estimates of wild population size vary between 1,000 and 10,000 individuals (King et al. 2020), and ongoing threats such as habitat loss and hunting further endanger the species (Schwitzer et al. 2019).

Although several studies have explored adult indris' ecology and feeding behavior (Pollock 1975a; Britt et al. 2003; Randrianarison et al. 2022), little is known about how these traits develop during infancy. Observing infant development in the wild is particularly difficult due to low visibility in rainforest environments and the species' canopy‐dwelling habits. However, understanding how infants transition from complete maternal dependence to independence, particularly in locomotion, diet, and social behavior, is essential both for understanding species ecology and informing conservation efforts. Early behavioral development is especially important in indris, which must learn to navigate the towering tree canopy and identify edible plants before becoming too heavy to be carried by their mothers. Previous reports provide mostly anecdotal descriptions of infant behavior (Pollock 1975b), and it remains unclear how maternal behavior influences developmental trajectories. For instance, indris may begin singing at 1 year of age (De Gregorio et al. 2021) or later (Pollock 1975a), and the timing of such milestones may vary across populations. Similarly, while co‐feeding and dietary overlap with the mother are likely important for social and nutritional development, their dynamics during infancy are still poorly documented. In this study, we systematically examine the behavioral and dietary development of wild indri infants over the first 2 years of life.

We aim to describe the progression toward independence in multiple domains, with a focus on locomotion, play, grooming, and feeding. Specifically, we predict that: (i) infants will become increasingly independent during the first year, shown by a reduction in maternal carrying and an increase in self‐initiated movement and play; (ii) Their diet will shift from maternal milk to a broader range of solid foods (e.g., leaves and fruits), increasing dietary diversity with age; (iii) Co‐feeding with the mother will decrease as infants begin to forage more independently. By addressing these questions, we aim to deepen our understanding of early development in indris and offer practical insights into the ecological needs of this species, with implications for conservation strategies and future attempts at ex‐situ management.

## 2 Materials and Methods

### 2.1 Ethic statement

The non‐invasive methods used for the data collection of this study adhere to the American Society of Primatologists (ASP) “Principles for the Ethical Treatment of Non‐Human Primates.” Field data collection protocols were reviewed and approved by Madagascar's Ministère de l'Environnement, de l'Ecologie et des Forêts on July 1, 2022, under Permit 2022:186/22/MEDD/SG/DGGE/DAPRNE/SCB.Re, and on March 17, 2023 under permit 084/23/MEDD/SG/DGGE/DAPPRNE/SCBE.Re. Field data collection protocols were also approved by GERP (Groupe d'Etude et de Recherche sur les Primates de Madagascar), the association overseeing research in the Maromizaha New Protected Area.

#### 2.1.1 Observations and Data Collection

We conducted behavioral observations from May 12, 2022, to July 23, 2023, in Maromizaha Forest (18°56'49"S‐48°27'33"E), a 2,150‐ha protected area in the Moramanga district of the Alaotra‐Mangoro region of Madagascar. Using the focal animal sampling method (Altmann 1974), we collected data on the behavior and feeding ecology of eight young individuals—five of which born in 2021 and three born in 2022—from eight habituated family groups (1MZ, 3MZ, 4MZ, 8MZ, 9MZ, 10MZ, 11MZ, 12MZ, Table 1). We noted behavioral transitions, the type of food ingested, along with the duration of each feeding session. Additionally, we recorded the type of food the mother was consuming during these feeding sessions. Each focal animal was observed for a maximum of five consecutive days, from approximately 6 a.m. to 1 p.m., covering peak activity hours and ensuring the group could be relocated the following morning. In total, observations were conducted over 67 days, resulting in 428.25 h of data. We monitored each family group and focal animal weekly, in rotation, to ensure equal data representation for each individual. One individual (Nusnus, Group 3) disappeared after only 2 days of observation, likely due to predation. Despite the limited data, it was included in the analysis because behaviors were classified by age class rather than by individual. We recognized the individuals through body size and natural pelage marks. For individuals born in 2022, identification relied on recognition of the mother, to whom they were still closely dependent. We assigned a precise date of birth to each individual through direct observation. When this was impossible, we assigned the 15th of the offspring's birth month as the birth date (De Gregorio et al. 2021).

**Table 1:** Name, social group, year of birth (YOB), and day of birth (DOB) of the study animals.

Name	Group	YOB	DOB
Fetsy	11MZ	2021	15 May
Nissa	12MZ	2021	1 June
Rio	1MZ	2021	15 June
Nusnus	3MZ	2021	15 June
Toky	8MZ	2021	15 June
Mofo	4MZ	2022	31 May
Rano	10MZ	2022	15 June
Bruno	9MZ	2022	5 Sept

The study monitored young individuals using an ethogram with 5‐min scans, recording 23 mutually exclusive behaviors (Table SM1) and entering ‘Not Available’ (NA) when it was not possible to record the behavior. Vigilance behavior was defined broadly to include both scanning for potential threats and non‐specific observation of the environment, as these categories could not always be reliably discriminated in young individuals. Negative interactions were defined as events in which an individual attacked and displaced a conspecific either to occupy its position in the tree (‘Negative interactions for a place’) or to gain access to its food resource (‘Negative interactions for feeding’).

Feeding sessions were also reported through 5‐min scans. We collected information on the consumed plant, type of food ingested, exact time, and total feeding duration (in minutes). When the mother was present, we collected the same data from her feeding session. Suckling behavior was considered to be the ingestion of the mother's milk. During the study, we could determine the sex of only one offspring (Fetsy, 11MZ, male) due to the absence of sexual dimorphism within the species (Pollock 1975b). Sex determination is only possible when the individual exhibits singing behavior characterized by a sex‐specific acoustic pattern (Giacoma et al. 2010). Before conducting the analyses, we categorized the monitored individuals into four age classes, *i.e*., 0‐3 months, 4–7 months, 10–15 months, and 16–20 months. The 8–9 months age class was excluded, as no young indris within this range were observed during our data collection.

#### 2.1.2 Statistical Analyses

− *Behavioral ontogeny*
  The behavioral sequences were analyzed using Beatrix‐0.9.22 (Friard and Gamba 2021), open‐source software that generates a flowchart code representing the behaviors' transitions. Moreover, this software performs a permutation test to show the statistical significance associated with the different transitions. We considered behavioral sequences for each age group, for which we calculated descriptive statistics. To validate the statistical significance of our data, we used the Random Permutation Test with 10,000 permutations. We applied a cut‐off of 5% of transitions after behavior. The resulting flow chart visually represents the transition probabilities between different behaviors in each age group, with bold arrows indicating significant transitions.
− *Feeding behavior*
  we considered 113 feeding sessions for the 0–3 month age class, 191 for the 4–7 month age class, 281 for the 10–15 month age class, and 363 for the 16–20 month age class. We used the software R (version 4.3.2, R Core Team 2023) to investigate which kind of plants indris consumed at these different month age classes. We calculated the total duration of each animal's daily feeding sessions and the total minutes it spent consuming each tree family. Within each month age class, we calculated the percentage of the overall consumption of each family by dividing the total amount of time that has been spent consuming a certain plant family by individuals of a specific month age class by the total amount of time that has been spent feeding by the individuals who belonged to that month age class.
  We did the same to understand which type of food individuals in the different month age classes consumed. The type of food included leaves, fruit, flowers, branches (bark and pith), mother's feces, and milk (suckling). We calculated the percentage of consumption of different types of food for the 4 month age classes by calculating daily percentage per individual by dividing the total amount of time that was spent consuming a certain type of food by the individuals who belonged to a month age class for the total amount of time that was spent feeding by the individuals who belonged to a month age class.
− *Co‐feeding*
  To investigate the presence of a co‐feeding relationship between the individuals and their mothers, we considered the feeding sessions in which they fed simultaneously, for a total of 495 feeding sessions. We calculated a co‐feeding ratio value for each feeding session as the ratio between the total number of daily feeding sessions where the animals ate the same plant and divided by the sum of that value plus the total number of daily feeding sessions where different plants were eaten. We then used a Generalized Linear Mixed Model (GLMM, *glmmTMB* package; Brooks et al. 2017), where the co‐feeding ratio was entered as the response variable, and the age of the offspring (in days) was used as a predictor. We checked the distribution of the response variable via the package *fitdistrplus* (Delignette‐Muller and Dutang 2015) and found that “beta” was a suitable theoretical distribution.

## 3 Results

### 3.1 Behavioral Ontogeny

− Age class 0–3 months
  In the first 3 months of life, infant indris were mostly carried ventrally by their mothers, with vigilance as the second most common behavior. Play made up 11.5% of behaviors, mainly individual or with the mother, and locomotion was limited to short distances (up to 1 m). One infant was observed moving more than 1 m away from its mother at 53 days old. In this age class, resting, feeding, and locomotion occurred at similar rates; grooming, scratching, yawning, and vocalizing were rare (Figure 1G). Grooming only occurred between mother and infant. Behavior transitions frequently involved carrying and vigilance, play and locomotion, and resting and carrying (Figure 1A). Less common were transitions involving grooming, scratching, and feeding. The full set of behavioral frequencies and transitions can be found in Tables 2, 3, and Figure SM1.
− Age class 4–7 months
  Between four and 7 months of age, infant indris began moving several meters away from their mothers, although falls were still common due to immature motor skills. Locomotion became the most frequent behavior, followed by vigilance and being carried, now exclusively on the back (Figure 1G). Self‐grooming and individual play were also common, with the first social play and grooming involving individuals other than the mother recorded at 128 days. Less frequent behaviors included feeding, scratching, and resting, while rare behaviors like yawning, geophagy, and marking were occasionally observed. Behavioral transitions were dominated by sequences involving locomotion, play, and vigilance, with some transitions, like between grooming and scratching or feeding and vigilance, occurring less often (Figure 1B). The full set of behavioral frequencies and transitions can be found in Tables 2, 3, and Figure SM2.
− Age class 10–15 months
  Between 10 and 15 months of age, vigilance became the most frequent behavior among infant indris, followed by locomotion, feeding, and self‐grooming.Social interactions and play became rare, while behaviors like scratching, resting, and grooming occurred with low frequency (Figure 1G). Several significant behavioral transitions were identified, particularly between feeding and locomotion, and between vigilance and locomotion. Less frequent transitions included those between resting, self‐grooming, and vigilance (Figure 1C). Rare behaviors such as geophagy and marking were also occasionally followed by locomotion. The full set of behavioral frequencies and transitions can be found in Tables 2, 3, and Figure SM3.
− Age class 16–20 months
  **Figure 1:**
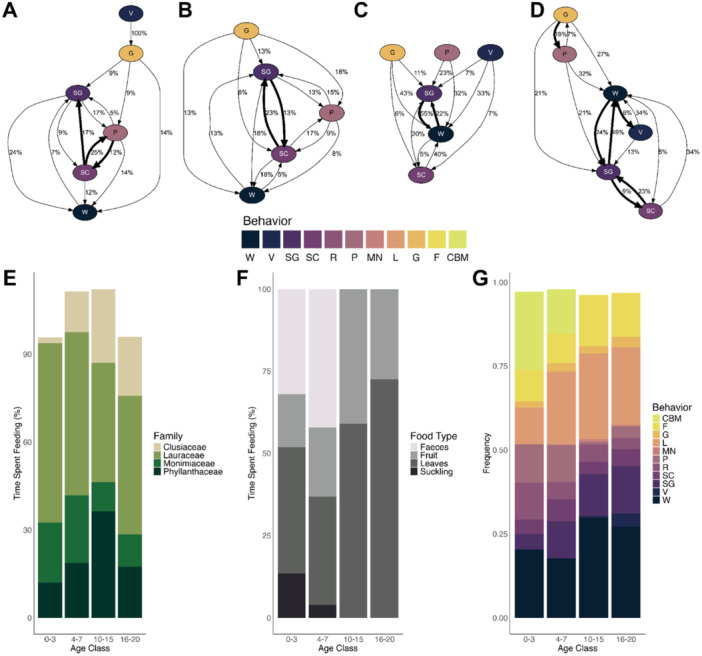
The variation in the diet and behavior of four different month age classes in wild indris (0–3, 4–7, 10–15, and 16–20 months) in the Maromizaha Forest, Madagascar. The first four panels display the behavioral transitions that occur at 0–3 months (A), 4–7 months (B), 10–15 months (C), and 16–20 months (D). Solid thick lines denote significant transitions. The remaining panels demonstrate the four tree families that are most commonly consumed (E) and the frequently ingested food types (F), and the 10 most common behaviors (G). W: vigilance, V: vocalizing, SG: self‐grooming, SC: scratching, R: resting, P: play; MN: marking by neck glands, L: locomotion, G: grooming, F: feeding, CBM: infant carried by mothers.
  **Table 2:** Behavioral frequencies of infant indris by age class.

Behavior	0–3 mo (%)	4–7 mo (%)	10–15 mo (%)	16–20 mo (%)
Feeding (F)	9.4	8.9	15.2	13.1
Sleeping (SL)	0.6	0.5	0.6	0.4
Resting (R)	10.9	5.2	5.1	3.4
Vigilance (W)	20.3	17.7	29.8	27.2
Locomotion (L)	11.0	21.6	25.5	23.2
Carried by mother (CBM)	23.3	13.3	—	—
Self‐grooming (SG)	4.6	11.1	12.4	14.1
Grooming (G)	1.8	2.5	2.3	3.1
Yawning (Y)	1.9	0.6	0.2	0.8
Scratching (SC)	4.3	6.4	3.8	5.0
Licking/sniffing (L/S)	0.1	0.1	0.3	< 0.1
Urination/defecation (U/F)	0.1	0.6	2.3	1.4
Marking with urine (MU)	—	—	—	0.1
Marking with feces (MF)	—	—	—	< 0.1
Marking by genital rubbing (MG)	—	< 0.1	< 0.1	—
Marking by neck glands (MN)	—	0.3	0.9	0.4
Geophagy (GE)	—	0.2	0.3	0.3
Vomiting/reingestion (V/R)	—	0.1	< 0.1	—
Playing (P)	11.5	11.0	0.7	3.4
Singing (S)	—	—	—	0.1
Vocalizing (V)	0.1	—	0.5	3.9
Neg. int. for place (NIP)	—	—	—	< 1
Neg. int. for food (NIF)	—	—	0.1	< 1
  **Table 3:** Notable behavioral transitions of infant indris by age class.

Age Class	Behavioral Transitions (*p* < 0.001 unless stated)
**0–3 mo**	CBM‐ > W (47.5%), W‐ > CBM (38.1%), L‐ > P (47.3%), P‐ > L (23.1%), R‐ > CBM (42.0%), CBM‐ > R (21.2%), SC‐ > P (25.0%, *p* = 0.001), P‐ > SC (11.5%), F‐ > P (20.3%, *p* = 0.001), W‐ > R (17.8%), SC‐ > SG (16.7%), W‐ > F (14.8%)
**4–7 mo**	U/F‐ > L (56.2%, *p* = 0.002), CBM‐ > W (48.1%), W‐ > CBM (30.9%), P‐ > L (42.7%), L‐ > P (29.3%), SL‐ > R (41.7%), F‐ > L (33.9%), SG‐ > L (37.5%), L‐ > SG (17.6%), R‐ > CBM (26.1%), CBM‐ > R (8.8%), F‐ > W (24.3%, *p* = 0.004), SC‐ > SG (22.6%), SG‐ > SC (13.1%), CBM‐ > F (13.8%, *p* = 0.001), W‐ > R (11.7%)
**10–15 mo**	GE‐ > L (75.0%, *p* = 0.004), F‐ > L (52.2%), L‐ > F (31.4%), L‐ > W (49.2%), W‐ > L (43.1%), SG‐ > W (54.6%), W‐ > SG (21.9%), U/F‐ > W (44.4%, *p* = 0.004), R‐ > W (43.3%), W‐ > R (7.0%, *p* = 0.001), R‐ > SG (21.0%, *p* = 0.002), SG‐ > R (8.4%, *p* = 0.001)
**16–20 mo**	NIP‐ > S (100%, *p* = 0.002), GE‐ > L (72.7%, *p* = 0.001), F‐ > L (48.9%), U/F‐ > L (45.8%), L‐ > W (45%), W‐ > L (37.5%), MU‐ > MF (40.0%), SG‐ > W (48.5%), W‐ > SG (24.0%), R‐ > W (45.5%), W‐ > R (6.7%), V‐ > L (33.9%), L‐ > F (29.3%), SC‐ > F (25.2%), SL‐ > G (25.0%, *p* = 0.001), SC‐ > SG (22.4%), SG‐ > SC (8.8%), G‐ > P (18.8%), F‐ > SC (9.7%), W‐ > V (5.9%), SG‐ > R (5.3%, *p* = 0.005)
  Between 16 and 20 months, vigilance remained the most frequent behavior, followed closely by locomotion, self‐grooming, and feeding (Figure 1G). Scratching, vocalizing, and playing occurred less frequently. Several significant behavioral transitions were observed, such as between locomotion and vigilance, and feeding and locomotion (Figure 1D). Notably, singing behavior emerged after negative interactions for the place on the tree, and geophagy frequently preceded locomotion. Other transitions, like self‐grooming to vigilance and resting to vigilance, also showed notable occurrences. Less common transitions included grooming and play, as well as vigilance and vocalization. The full set of behavioral frequencies and transitions can be found in Tables 2, 3, and Figure SM4.

### 3.2 Feeding Behavior

− Age class 0–3 months
  Infants between zero and 3 months of age consumed 13 different plant families (Figure SM5). Lauraceae were the most frequently eaten (43.74% of the time, Figure 1E), followed by Annonaceae (20.09%), Monimiaceae (10.05%), and Phyllanthaceae (8.81%), while Clusiaceae were consumed only for 0.31% of the time. At this age, leaves were the most consumed type of food (49.09%), while fruits were eaten 15.14% of the time. Maternal milk was also consumed 19.74% of the time, and seven events of the mother's feces consumption were observed (Figure 1F).
− Age class 4–7 months
  Lauraceae was the most consumed plant family also in this month age class (60.73%), followed by Annonaceae (14.09%), Clusiaceae (8.14%), Monimiaceae (6.07%), and Phyllanthaceae (5.25%) (Figure 1E). Indris, between four and 7 months, consumed a total of 13 different plant families (Figure SM5). Leaves remained the most common type of food, accounting for 43.59% of the feeding time. However, the fruit percentage rose to 27.09%, while the time spent feeding on maternal milk was 10.62% (Figure 1F). Mother's feces consumption was also observed once.
− Age class 10–15 months
  Indris belonging to this month age class consumed Lauraceae for 37.62% of the feeding time, a slightly lower value when compared to the previous month age classes. In addition, Phyllanthaceae consumption showed an upward trend and reached 25.46% of the time, followed by Clusiaceae (16.13%) and Monimiaceae (7.27%) (Figure 1E). They consumed a total of 15 different plant families (Figure SM5). No events of feeding on maternal milk or feces were recorded. Leaves resulted again as the most eaten type of food (54.68%), followed by fruits (30.25%) and branches (12.66%) (Figure 1F).
− Age class 16–20 months
  Lauraceae was once more the most consumed plant family (46.64% of the time, Figure 1E). Clusiaceae followed with 15.57%, followed by Phyllanthaceae (10.40%) and Monimiaceae (7.01%) (Figure 1E). They consumed a total of 12 different plant families (Figure SM5). Again, no consumption of milk or feces was observed. The individuals ate leaves for 66.24% of the time (Figure 1F), fruit for 25.01%, and branches for 7.10%.

### 3.3 Co‐Feeding

**Figure 2:**
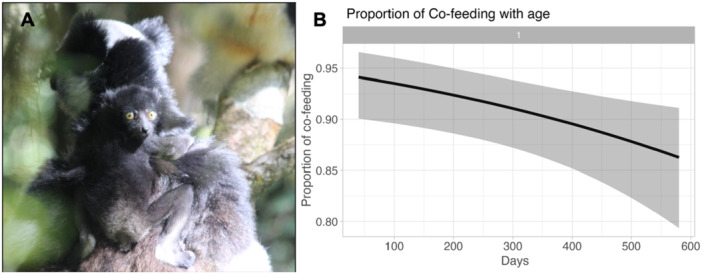
(A) An infant indri (50 days of age) carried by its mother. (B) Effect of age (in days) on the proportion of feeding sessions in which mothers and offspring consumed the same plant. Shaded areas indicate confidence intervals. The gray area represents 95% confidence interval.

We found that, out of 495 feeding sessions, in 438 of them, the mother and young ate the same plant, while in the other 57, they did not. We observed a total of 81 co‐feeding events for the age class 0–3 months, for which the mean time spent eating the same plant as the mother was 5.69 min. Individuals from the age class 4–7 months spent an average of 5.30 min co‐feeding with the mother, with a total of 146 observations of this behavior. The average time spent co‐feeding for the age class 10‐15 months was 14.78 min with 73 observations, while for the age class 16‐20 months, these values were 17.57 min and 184 observations. Moreover, the model regarding the co‐feeding relationship showed that, as the young's age increases, the proportion of co‐feeding with the mother declines (Estimate = ‐0.002, SE = 0.0006, *p* = 0.009, Figure 2).

## 4 Discussion

This study enhances our understanding of behavior and diet in young indris by documenting key developmental milestones during their first year of life through longitudinal observations. By testing specific hypotheses on the progression toward independence, dietary diversification, and co‐feeding dynamics, we provide a detailed baseline of early ontogeny in a Critically Endangered species. These findings are not only valuable for improving our general knowledge of primate development but also have practical applications: they can inform conservation strategies, guide future comparisons with indris living in disturbed habitats, and help explain past failures in captive breeding by highlighting dietary and behavioral needs during infancy.

### 4.1 Infant Indri Ontogeny Across Age Classes

We found that infants were predominantly carried by their mothers until 3 months of age, with carrying decreasing as locomotion increased between four and 7 months. During this period, we observed frequent behavioral transitions from feeding to locomotion, suggesting a shift from exclusive nursing to independent foraging on solid foods (Figure 1A). Consistent with Pollock (1975b), carrying typically ceases by the 8th–9th month. Resting, highest in the first month, declined with age and then stabilized, while yawning peaked in the 0–3‐month class and subsequently fluctuated (Figure 1G).

As infants grow, time spent being carried and resting declines, giving way to increased play, particularly between 0 and 3 and 4–7 months (Figure 1G). During these stages, play correlates strongly with locomotion and feeding, suggesting its key role in learning to move and identify edible items. Scratching behavior, often linked to stress, also peaks during this period (Maestripieri et al. 1992; Troisi 2002; Palagi and Norscia 2011). Infants were frequently seen manipulating leaves, branches, and fruits before consuming them. The play–locomotion correlation remains high at 4–7 months, highlighting ongoing motor skill development needed for adult leaping. Moreover, in this class age, anecdotal falls were observed (twice for Mofo and once for Rano), suggesting ongoing motor skill development. Play decreases at 10–15 months but rises slightly at 16–20 months, when transitions between feeding, locomotion, vigilance, and self‐grooming become more common (Figure 1B). At this stage, play follows locomotion, indicating a more complex behavioral repertoire.

Pollock (1975b) highlighted the importance of the second to third month for motor skill development in indris, with independent movement typically achieved by nine to ten months. Our results suggest that once infants acquire key skills for locomotion and feeding, the functional need for play decreases, explaining the drop in play frequency in the 10–15‐month age class. This pattern mirrors findings in *Alouatta guariba clamitans*, where play declined by 50% after a peak at 5–6 months (Podgaiski and Jardim 2009), and in *Trachypithecus leucocephalus*, where non‐social play peaked at 5 months and declined thereafter, while social play increased with age (Yang et al. 2022). It is likely that, as young primates develop motor skills and begin independent foraging, the need for non‐social play decreases, while social play becomes more relevant for practicing interactions and building relationships. This shift likely reflects growing social demands and the importance of learning affiliative behaviors for group integration.

We found that in the 10–15 month class, vigilance behavior increases markedly (Figure 1G), reflecting the young's growing need to detect potential threats independently. From 10 months onward, vigilance is strongly associated with locomotion, suggesting that juveniles scan for danger while moving, and with vocalizations, indicating early signs of alarm‐calling. Our results are in line with previous work (Pollock 1975b) showing that indris reach independence around 12 months, when suckling ceases. This timing is earlier than in some primates, such as *Trachypithecus leucocephalus* (19–21 months; Zhao et al. 2008) and wild Hanuman langurs (25 months; Borries and Koenig 2000), but later than in species like wild vervets, where weaning occurs at 6 months (Struhsaker 1971). Compared to other lemur species such as *Lemur catta* and *Eulemur flavifrons*, which reach independence around four (Gould 1990) and 6 months, respectively (Volampeno et al. 2011), and benefit from some form of alloparental care, indris appear to rely exclusively on maternal care. In black‐and‐white ruffed lemurs (*Varecia variegata*), which exhibit alloparental care too, infants have been observed to reach adult size as early as 6 months of age, although some variability exists (Morland 1990). In our observations, we never recorded paternal involvement, such as infant carrying. Although the exact reason for this interspecific difference in developmental timing is not yet clear, the level of alloparental care might play a role in accelerating or delaying infant independence.

In our study, social behaviors such as collar neck marking and genital rubbing emerged early, particularly in the 4–7‐month class. As the indris matured, urination and defecation increased with age, with urine marking observed from 10 to 15 months and fecal marking from 16 to 20 months.

### 4.2 Development of Social Interactions

The swift emergence of social behaviors in indris highlights the importance of early social development for navigating mother–infant relationships and establishing broader social ties within the group. We found that grooming gradually increased with age, reflecting developmental changes in its function (Pollock 1975b). Early on, grooming occurred only between mother and infant, but as self‐grooming developed, especially from 4 to 7 months, maternal grooming declined (Figure 1G). From this age, infants also began engaging in allogrooming and social play, likely to foster social bonds. These behaviors appeared earlier than Pollock reported, possibly due to individual variation or differences in mother–infant dynamics. As suggested by Altmann (1980), less tolerant mothers may accelerate independence. Negative interactions, including food‐related conflicts, emerged around 10 months of age, while spatial disputes became apparent between 16 and 20 months, suggesting that such conflicts were largely absent before 10 months. Volampeno et al. (2011) observed that young *Eulemur flavifrons* began engaging in social play during the seventh week of life, whereas other social behaviors, such as allogrooming and fighting, emerged around the 28th week. Additionally, young *Lemur catta* were observed playing with their mothers for the first time in the third week of life, and with other conspecifics by the sixth week. They also began displaying dominance behaviors, such as cuffing and supplantation, by the 15th week, although such behaviors were observed only rarely (Gould 1990). These differences among lemur species could be related to differences in social organization and group size, with indris living in small family groups while others reside in large multi‐male, multi‐female groupings (Randriatahina and Roeder 2012; Sauther and Sussman 1993).

Like social and feeding behaviors, vocal development in indris follows a protracted trajectory, with early calls supporting basic interactions and later stages culminating in the acquisition of singing. Vocal behavior is infrequent until the 16‐20 class (Figure 1G), and vocalizations are emitted in various contexts, such as contact between individuals and predator warning. Moreover, singing behavior was observed only after 16 months of age. This finding suggests, again, individual variability in behavioral development, as a previous study found that indris started to sing at 11.88 months old for females and 14.74 months for males (De Gregorio et al. 2021). Such variation could be influenced by multiple factors, including group composition: when many individuals sing, opportunities for others to participate, especially young ones, may be limited. Additionally, it may depend on the degree of parental stimulation, such as brief solo bouts directed at infants to promote vocal engagement (De Gregorio et al. 2022).

### 4.3 Development of Feeding Behavior

Ontogenetic changes in feeding behavior are crucial for the transition from dependence on maternal care to nutritional independence, and may involve both unique developmental strategies and gradual dietary diversification. We documented early‐occurring peculiar feeding behaviors. Regurgitation and reingestion appeared in the 4–7 month class, marking the third such observation in infant indris (Randrianarison et al. 2022), possibly linked to plant secondary compounds. Geophagy also emerged between 4 and 7 months, likely aiding in toxin neutralization, pH regulation, mineral intake, and digestion (Pebsworth et al. 2019; Borruso et al. 2021). Lastly, coprophagy occurred between 52 and 127 days of age, peaking around 70 days, and may serve to transfer maternal microbiota, as seen in Coquerel's sifakas' infants (McKenney et al. 2015). Moreover, this is in line with recent evidence on how social group membership shapes gut microbiome composition in the indris, with the highest transmission observed between mothers and their offspring (Labisa‐Morais et al. 2025). Coprophagy has also been documented in ring‐tailed lemurs (*Lemur catta*), who consumed feces from humans, dogs, and cattle, possibly as a strategy to supplement nutrients, particularly in older individuals with dental problems (Fish et al. 2007). In contrast, we found no such behavior in adult indris, suggesting that in infants, coprophagy may support microbiota development, in line with studies on other mammals, showing that coprophagy plays a role in seeding or restoring gut microbiota during early development or periods of illness (Bo et al. 2020; Osawa et al. 1993). This also aligns with the presence of geophagy and regurgitation/reingestion in *Indri indri*, especially during the dietary shift from milk to foliage. Notably, neither suckling nor coprophagy was observed after 7 months of age.

### 4.4 Development of Diet

We found age‐related differences in the percentage of time spent eating various plant families and food types (Figure 1E,F). Overall, we recorded the consumption of 18 plant families, a slightly lower number than the 24 reported by Randrianarison et al. (2022), likely due to their longer sampling period (9 years). It's also possible that younger indris had not yet begun consuming certain plant families eaten by adults, a hypothesis supported by Britt et al. (2003), who reported 22 plant families over a 12‐month period.

Overall, feeding patterns appear broadly consistent across studies, with some variation likely driven by site‐specific plant availability and sampling differences. Lauraceae were consistently the most consumed plant family across all age classes (Figure 1E). In contrast, Clusiaceae showed a marked increase in consumption with age: they accounted for only 0.31% of feeding time in the first age class but rose to 16.13% and 15.57% in the third and fourth classes, becoming the second most consumed family during the second and third classes. Conversely, Monimiaceae exhibited a slight decline, decreasing from 10.05% in the first age class to 6.07% in the second, and remained relatively stable thereafter. Similar to Clusiaceae, Phyllanthaceae consumption increased with age, from 8.81% between zero and 3 months to a peak of 25.46% between 10 and 15 months, before dropping to 10.40% in the final age class, at which point Clusiaceae once again became the second most consumed family. These patterns are broadly consistent with Randrianarison et al. (2022), who identified Lauraceae, Monimiaceae, and Phyllanthaceae as the most consumed families, though our study recorded a higher intake of Clusiaceae than Monimiaceae. Similarly, Britt et al. (2003) found Lauraceae and Clusiaceae to be the top two plant families consumed, with Myristicaceae ranking third, yet this family was not observed in our study, likely due to differences in plant availability across study sites, as the flora composition of Maromizaha may differ from other areas.

In our study, the types of food consumed varied across age classes (Figure 1F). Leaves were the most consistently consumed food item throughout development, aligning with previous findings on this species (Randrianarison et al. 2022; Britt et al. 2003; Powzyk and Mowry 2003; Pollock 1975b). Notably, fruit consumption increased from 15.14% to 27.09% between the first and second age classes and remained relatively stable thereafter. Although seasonal availability of fruits and flowers was considered (Pollock 1975b; Randrianarison et al. 2022), our observations suggest that consumption patterns were not strictly tied to seasonal peaks. For instance, flowers were consumed more frequently between October and December, outside their typical peak season. This supports previous findings (Randrianarison et al. 2022; Britt et al. 2003) showing limited seasonal influence on food type consumption. Thus, the gradual increase in fruit and leaf intake after 10 months likely reflects normal dietary development. Suckling was last observed at 204 days of age, earlier than Pollock's report of around 1 year (1975b). However, Pollock's observation was based on a single individual, suggesting that individual variability or other ecological factors (e.g. Pollock's research site did experience selective logging prior to his field research) may account for this difference.

### 4.5 Co‐Feeding

Our model revealed a co‐feeding relationship between mothers and offspring that declined with age (Figure 2). This result is consistent with findings in *Macaca fuscata* (Ueno 2005) and *Lemur catta* (O'Mara and Hickey 2012), although the latter species showed this behavior less frequently. Pollock (1975b) suggested feeding synchrony in indris, but not specifically within the mother‐offspring dyad. In our study, the ratio of co‐feeding between mothers and young decreased with age, similar to the trends observed in Japanese macaques (Ueno 2005). However, our results diverged from those of O'Mara and Hickey (2012), who found that younger *Lemur catta* exhibited higher co‐feeding rates, with sub‐adults showing the highest. In contrast, Indri indri showed a consistent decline in co‐feeding. Although the average time spent co‐feeding was higher for the age classes 10–15 and 16–20 months, this is only due to an increase in duration of feeding sessions, as the proportion of feeding time during which this behavior was observed declined consistently with age. This behavior may facilitate social learning, enabling young indris to learn about food recognition, foraging locations, and processing methods (O'Mara and Hickey 2012). Given that indris consume certain plants known to contain high levels of secondary metabolites, including potentially toxic compounds (Powzyk and Mowry 2003; Randrianarison et al. 2022), social learning likely plays a crucial role in teaching infants which items are safe to eat and how to handle them. As highlighted by Van Boekholt et al. (2021), social learning thrives when the model individual is tolerant and consistently available, which is often true for mothers. In primates, this results in high overlap of learned skills between mothers and infants through vertical social transmission.

## 5 Conclusions

Our research provides key insights into the development of *Indri indri* infants, focusing on their behavioral and dietary progression. Infants gradually become more independent, with play comprising an important element of exploring the environment, developing locomotion, and learning about food. Social interactions shift over time, with grooming evolving from maternal care to social bonding, and co‐feeding with the mother decreasing. Indris show early independence compared to other primates, but later than other lemur species, stopping milk consumption by 7 months and feeding independently by 14 months. The presence of coprophagy and geophagy may be vital for proper feeding behavior and gut microbiome development. These findings contribute to the conservation of this Critically Endangered species by stressing the importance of age‐specific diets and social settings essential for successful captive breeding.

## Author Contributions

**Giada Brunod:** data curation (lead), investigation (lead), visualization (equal), writing – original draft (equal), writing – review and editing (equal). **Federica Dellepiane:** formal analysis (lead), investigation (lead), visualization (equal), writing – original draft (equal), writing – review and editing (equal). **Daria Valente:** writing – review and editing (equal). **Valeria Ferrario:** writing – review and editing (equal). **Filippo Carugati:** writing – review and editing (equal). **Valeria Torti:** writing – review and editing (equal). **Jonah Ratsimbazafy:** writing – review and editing (equal). **Cristina Giacoma:** funding acquisition (equal), project administration (equal), writing – review and editing (equal). **Marco Gamba:** methodology (equal), supervision (equal), validation (equal), visualization (lead), writing – review and editing (equal). **Chiara De Gregorio:** conceptualization (lead), data curation (equal), formal analysis (lead), investigation (equal), methodology (equal), project administration (lead), supervision (lead), validation (lead), visualization (equal), writing – original draft (lead), writing – review and editing (lead).

## Conflicts of Interest

The authors declare that the research was conducted without any commercial or financial relationships that could potentially create a conflict of interest.

## Supporting information

Supportig Information

Supportig Information

Supportig Information

Supportig Information

Supportig Information

**Fig. SM1:** Overall behavioral transitions occurring in indris aged 0‐3 months. Solid thick lines denote significant transitions. **Fig. SM2:** Overall behavioral transitions occurring in indris aged 4‐7 months. Solid thick lines denote significant transitions. **Fig. SM3:** Overall behavioral transitions occurring in indris aged 10‐15 months. Solid thick lines denote significant transitions. **Fig. SM4:** Overall behavioral transitions occurring in indris aged 16‐20 months. Solid thick lines denote significant transitions. **Fig. SM5:** Time spent feeding for all the plant families per age class.

**Table SM1:** Ethogram of observed behaviors and corresponding symbols.

## Data Availability

The data that support the findings of this study are available from the corresponding author upon reasonable request.
