# Supplementary material for: Ontogeny of Diet and Behavior of a Wild, Critically Endangered Lemur (Indri indri)
Source: Am J Primatol. 2025 Dec 18;87(12):e70107. doi: 10.1002/ajp.70107 (PMC12712748; doi:10.1002/ajp.70107)
Supplement: Supplementary file 6 — Fig. SM1: Overall behavioral transitions occurring in indris aged 0‐3 months. Solid thick lines denote significant transitions. Fig. SM2: Overall behavioral transitions occurring in indris aged 4‐7 months. Solid thick lines denote significant transitions. Fig. SM3: Overall behavioral transitions occurring in indris aged 10‐15 months. Solid thick lines denote significant transitions. Fig. SM4: Overall behavioral transitions occurring in indris aged 16‐20 months. Solid thick lines denote significant transitions. Fig. SM5: Time spent feeding for all the plant families per age class. [file AJP-87-e70107-s005.docx]

**Captions _ supplementary figures _ Brunod et al.**

Fig. SM1: Overall behavioral transitions occurring in indris aged 0-3 months. Solid thick lines denote significant transitions.

Fig. SM2: Overall behavioral transitions occurring in indris aged 4-7 months. Solid thick lines denote significant transitions.

Fig. SM3: Overall behavioral transitions occurring in indris aged 10-15 months. Solid thick lines denote significant transitions.

Fig. SM4: Overall behavioral transitions occurring in indris aged 16-20 months. Solid thick lines denote significant transitions.

Fig. SM5: Time spent feeding for all the plant families per age class.
