# Supplementary material for: Ontogeny of Diet and Behavior of a Wild, Critically Endangered Lemur (Indri indri)
Source: Am J Primatol. 2025 Dec 18;87(12):e70107. doi: 10.1002/ajp.70107 (PMC12712748; doi:10.1002/ajp.70107)
Supplement: Supplementary file 7 — Table SM1: Ethogram of observed behaviors and corresponding symbols. [file AJP-87-e70107-s006.docx]

| **Behavior** | **Symbols** |
| --- | --- |
| Feeding | F |
| Sleeping | SL |
| Resting | R |
| Vigilance | W |
| Locomotion | L |
| Carried by mother | CBM |
| Self-grooming | SG |
| Grooming | G |
| Yawning | Y |
| Scratching | SC |
| Licking or sniffing | L/S |
| Urination or defecation | U/D |
| Marking by urine | MU |
| Marking by feces | MF |
| Marking by genital rubbing | MG |
| Marking by neck glands | MN |
| Geophagy | GE |
| Vomiting and reingestion | V/R |
| Playing | P |
| Singing | S |
| Vocalizing | V |
| Negative interactions for a place | NIP |
| Negative interactions for feeding | NIF |
| Not Available | NA |

Table SM1. Ethogram of observed behaviors and corresponding symbols.
